# Supplementary material for: Vicia faba SV channel VfTPC1 is a hyperexcitable variant of plant vacuole Two Pore Channels
Source: eLife. 2023 Nov 22;12:e86384. doi: 10.7554/eLife.86384 (PMC10665017; doi:10.7554/eLife.86384)
Supplement: Figure 12—source data 1. [file elife-86384-fig12-data1.pdf]

Figure 12—source data 1. TPC1-dependent lifetime of the post-stimulus depolarization phase.

Current stimulus (pA)	VfTPC1 $t_{plateau}$ (s)	AtTPC1-triple mutant E457/E605A/D606N $t_{plateau}$ (s)			
	Exp. 1-6	Exp. 1-3	Exp. 4	Exp. 5	Exp. 6
10	> 10.0	> 10.0	0.28	0.48	0.04
30	> 10.0	> 10.0	> 10.0	0.59	0.04
70	> 10.0	> 10.0	> 10.0	> 10.0	0.09
150	> 10.0	> 10.0	> 10.0	> 10.0	0.23
300	> 10.0	> 10.0	> 10.0	> 10.0	0.40
500	> 10.0	> 10.0	> 10.0	> 10.0	2.70
1000	> 10.0	> 10.0	> 10.0	> 10.0	> 10.0

Current stimulus (pA)	AtTPC1 wild type $t_{plateau}$ (s)						
	Exp. 1	Exp. 2	Exp. 3	Exp. 4	Exp. 5	Exp. 6	mean ± SE (n = 6)
10	0.05	0.05	0.06	0.02	0.12	0.05	**0.06 ± 0.13**
30	0.08	0.06	0.12	0.04	0.25	0.09	**0.11 ± 0.03**
70	0.10	0.07	0.15	0.05	0.26	0.12	**0.13 ± 0.03**
150	0.14	0.08	0.22	0.05	0.28	0.21	**0.16 ± 0.04**
300	0.19	0.08	0.31	0.06	0.36	0.28	**0.21 ± 0.05**
500	0.38	0.08	0.40	0.06	0.48	0.45	**0.31 ± 0.08**
1000	0.55	0.09	0.53	0.09	0.62	0.68	**0.42 ± 0.11**

Exp. 1 to 6 each represent a current clamp experiment with an individual vacuole.
