# Supplementary material for: Vicia faba SV channel VfTPC1 is a hyperexcitable variant of plant vacuole Two Pore Channels
Source: eLife. 2023 Nov 22;12:e86384. doi: 10.7554/eLife.86384 (PMC10665017; doi:10.7554/eLife.86384)
Supplement: Supplementary file 2. [file elife-86384-supp2.docx]

**Supplementary File 2. Primer sequences for subcloning and side-directed mutagenesis.**

| **Name** | **Sequence (5'- 3')** |
| --- | --- |
| AtTPC1^WT^ user fwd | GGCTTAAUATGGAAGACCCGTTGATTGG |
| AtTPC1^WT^ user rev | GGTTTAAUTCATGTGTCAGAAGTGGAACACTC |
| AtTPC1^D240A^ fwd | AGGCCACUCAGCAGGGCCTCACGGTC |
| AtTPC1^D240A^ rev | AGTGGCCUCAAACATAACAAAAGCAATCCAA |
| AtTPC1^D454A^ fwd | ACGCTTGCTAUCGAAGAAAGCTCGGCTCAG |
| AtTPC1^D454A^ rev | ATAGCAAGCGUTGTTTCAACAACGACAGCAA |
| AtTPC1^E457N^ fwd | AAACAGCUCGGCTCAGAAGCCATGG |
| AtTPC1^E457N^ rev | AGCTGTTUTCGATATCAAGCGTTGTTTCAA C |
| AtTPC1^E528A^ fwd | AGCATGGAUCCGGTACCTTCTCCTGGC |
| AtTPC1^E528A^ rev | ATCCATGCUCCATTTGAGAAGAAAGTATTCTCG |
| AtTPC1^E605A^ fwd | ATTGGCTGCGGAUGACTACCTTTTGTTCAAC |
| AtTPC1^E605A^ rev | ATCCGCAGCCAAUTCGGTTTCAAA |
| AtTPC1^D606N^ fwd | AGAGAATGACUACCTTTTGTTCAACTTCAAT |
| AtTPC1^D606N^ rev | AGTCATTCTCUGCCAATTCGGTTTCAAAGAG |
| VfTPC1^WT^ user fwd | GGCTTAAUATGACGGAGCCTCTACTCAGAG |
| VfTPC1^WT^ user rev | GGTTTAAUCCTGTACTGGAAGGATGATTTTGACAAA |
| LjTPC1^WT^ user fwd | GGCTTAAUATGGAACCTCTGCTCAGAGGCGAAAGCAGTG |
| LjTPC1^WT^ user rev | GGTTTAAUTTATGCATTGGAAGGCTGATCTTGACACAACTCAG |
| VfTPC1^A607E/N608D^ fwd | AGAGGATGACUATTTACTTTTTAATTTCAATG |
| VfTPC1^A607E/N608D^ rev | AGTCATCCTCUGCAAGGTCTGTGGCCTCCA |
| VfTPC1^N459E^ fwd | AAGAGAGTTCUGCTCAAAAGGCTTGG |
| VfTPC1^N459E^ rev | AGAACTCTCTUCTATGTCGAGCGTTGTCTCAA |

fwd = forward primer, rev = reversed primer
