# Supplementary material for: Vicia faba SV channel VfTPC1 is a hyperexcitable variant of plant vacuole Two Pore Channels
Source: eLife. 2023 Nov 22;12:e86384. doi: 10.7554/eLife.86384 (PMC10665017; doi:10.7554/eLife.86384)
Supplement: Supplementary file 3. [file elife-86384-supp3.docx]

**Supplementary File 3**

Details of the method used for computational simulation of electrical excitability shown in Figure 10.

**Computational simulation of electrical excitability at the tonoplast**

The basis of a transient electrical signal is the cable equation. Compared to an axon or the plant phloem, the membrane surface of the vacuole of a plant cell is very small. Therefore, on the time scale of the observed action potential, spatial equilibration across the tonoplast can be considered as quasi-instantaneous. In this case, the cable equation is simplified to:

$\frac{\partial}{\partial t}V\left( t \right)=\frac{1}{C_{M}}\left[ J_{Stim}\left( t \right)-\sum_{i} g_{i}\cdot p_{i}\left( t,V\left( t \right) \right)\cdot\left[ V\left( t \right)-E_{i} \right] \right]$ (Eq. 1)

The different parameters have the following meanings: (1) *V*(*t*): tonoplast voltage at time *t*; unit: mV. (2) *i*: ion species to be considered. (3) *E_i_*: equilibrium voltage for ion species *i*; unit: mV; (4) *g_i_*: maximum conductance for ion species *i*; unit: pS⋅µm^-2^; (5) *p_i_*(*t,V*(*t*)): ’open channel probability’ = activity of the conductance; 0≤*p_i_*≤1; (6) *J_Stim_*: external stimulus to excite the system; unit: fA⋅µm^-2^; (7) *C_M_*=*C_m_*/*A*: specific membrane capacity; unit: pF⋅µm^-2^; *C_m_*: membrane capacity of the vacuole; unit: pF. The following re-definitions remove the redundancy in the system-specific parameters: (1) $\varepsilon_{Stim}\left( t \right)=\frac{J_{Stim}\left( t \right)}{C_{M}}$ (unit: mV⋅s^-1^) is a constant describing the applied external stimulus. (2) $\varphi_{i}=\frac{g_{i}}{C_{M}}$ (unit: s^-1^) are channel specific constants that depend on the number of channels and the single channel conductance. With these definitions, the equation is:

$\frac{\partial}{\partial t}V\left( t \right)=\varepsilon_{Stim}\left( t \right)-\sum_{i} \varphi_{i}\cdot p_{i}\left( t,V\left( t \right) \right)\cdot\left[ V\left( t \right)-E_{i} \right]$ (Eq. 2)

To solve this equation numerically, the differential is approximated by a difference:

$\frac{\partial}{\partial t}V\left( t \right)\to\frac{V\left( t+\Delta t \right)-V\left( t \right)}{\Delta t}$ (Eq. 3)

In addition, the *t*-dimension is discretized in *M* points (index *m*= 0…*M*) with the interval of Δ*t* between two neighboring points: $V_{m}=V\left( t \right)$; $V_{m+1}=V\left( t+\Delta t \right)$; ${pi}_{m}=p_{i}\left( t,V\left( t \right) \right)$. With

$a_{m}=\Delta t\cdot\sum_{i} \varphi_{i}\cdot{pi}_{m}$, (Eq. 4)

$b_{m}=\Delta t\cdot\sum_{i} \varphi_{i}\cdot{pi}_{m}\cdot E_{i}$, (Eq. 5)

$\varepsilon_{m}=\Delta t\cdot\varepsilon_{Stim}\left( t \right)$, (Eq. 6)

the differential equation 2 converts into a linear equation that can be solved by iteration starting at $t=0$ with $V_{0}=V\left( 0 \right)$: $V_{m+1}=\left( 1-a_{m} \right)\cdot V_{m}+\varepsilon_{m}+b_{m}$ (Eq. 7)

**Mathematical description of channels and transporters in the vacuolar membrane**

*Background conductance*

The background conductance, which is dominated by proton pump activity, repolarizes the membrane voltage after electrical excitation. The background current (*I_BG_*) has been described by: $I_{BG}=I_{max}\cdot\frac{1-exp\left\{ -1.0\cdot\left( V-V_{0} \right)\cdot\frac{F}{RT} \right\}}{1+exp\left\{ -1.0\cdot\left( V-V_{0} \right)\cdot\frac{F}{RT} \right\}}$ (Eq. 8)

The parameter *V_0_* denotes the voltage, at which the background current is zero. For the simulations in this study we chose $V_{0}=-60 mV$. *I_max_* is the maximum current at positive voltages.

*TPC1 conductance*

The time- and voltage-dependent cation channel TPC1 imparts excitability to the vacuolar membrane. Its delayed-activating behavior can be described mechanistically by four independent gates of two different types following the gating schemes $O_{1} \begin{matrix} \underset{\leftarrow}{a1} \\ \overset{\to}{d1} \end{matrix} C_{1}$ and $O_{2} \begin{matrix} \underset{\leftarrow}{a2} \\ \overset{\to}{d2} \end{matrix} C_{2}$ with the rate constants *a_1_*, *a_2_*, *d_1_*, and *d_2_* for activation and deactivation, respectively: *a*_1_= s^‑1^ × exp[0.45 × *V*×*F*/(*RT*) - 0.26 × ln(α)], *d*_1_= s^‑1^ × exp[‑0.81 × *V*×*F*/(*RT*) + 0.26 × ln(α) + 1.84], *a*_2_= s^‑1^ × exp[0.5 × *V*×*F*/(*RT*) – 0.26 × ln(α) – 0.4], *d*_2_= s^‑1^ × exp[‑0.5 × *V*×*F*/(*RT*) + 0.26 × ln(α) + 3.0]. To simulate TPC1s with different gating features, the parameter α has been set to 0.002, 0.01, and 0.05. Assuming that K^+^ is the predominant permeating ion, the current through TPC1-type channels can be mathematically described by:

$I_{TPC}=\sigma_{TPC}\cdot p_{TPC}\cdot\left( V-E_{K} \right)$ (Eq. 9)

where *σ_TPC_* is the maximum membrane conductance of the TPC1 channel, which depends on the single channel conductance and the number of channels, and *p_TPC_* is the voltage-dependent open probability of the channels. *σ_TPC_* has been estimated from experimental data: Under standard symmetrical conditions (*E_K_*= 0 mV), at a voltage of +100 mV, we measured a steady-state current density of *I_ss_*/*C_m_*= 500 pA/pF, corresponding to a current of *I_TPC_*= 10 nA for a vacuole with a membrane capacity of *C_m_*= 20 pF. So, *σ_TPC_*= *I_TPC_*/*V*= 10 nA/100 mV = 100 nS. This value could be used to determine the parameter *ϕ_TPC_* needed for equations 2, 4, and 5: *ϕ_TPC_* = *g_TPC_*/*C_M_* = *σ_TPC_*/*A* × *A*/*C_m_* = 100 nS/20 pF = 5000×s^-1^, where *A* is the surface of the membrane.

*TPK conductance as a security valve at very positive voltages*

TPK channels were simulated as voltage-independent K^+^-selective channels. The current through TPK channels can be expressed as $I_{TPK}=\sigma_{TPK}\cdot\left( V-E_{K} \right)$ (Eq. 10)

where *σ_TPK_* is the conductance of this channel type in the membrane. *σ_TPK_* is stimulated in a TPC1-dependent manner. To simulate this effect, we chose the following possible scenario: *σ_TPK_* is activated proportionally to the current flowing through TPC1 and decays in a voltage-dependent manner *d*~exp(‑3×*V*×*F*/(*RT*)) with very low inactivation rates at positive voltages and increasing inactivation rates at more negative voltages. Because the parameter *ϕ_TPK_* (used in equations 2, 4, and 5) is proportional to *σ_TPK_*, *ϕ_TPK_* = *g_TPK_*/*C_M_* = *σ_TPK_*/*A* × *A*/*C_m_ = σ_TPK_*/*C_m_*, the transient activation can be directly modeled for *ϕ_TPK_*.

*Stimulus*

The capacity of a typical vacuolar membrane was *C_m_*= 20 pF. The stimulus current in the simulations was 300 pA ≤ *I_stim_*≤ 1000 pA applied for 100 ms. From these values, the parameter *ε_Stim_*(*t*) for equations 2 and 6 can be deduced: *ε_Stim_*(*t*) = *J_Stim_*(*t*)/*C_M_* = *I_Stim_*/*A* × *A*/*C_m_* = *I_Stim_*/*C_m_* → 15 *V*/*s* ≤ *ε_Stim_* ≤ 50 *V*/*s*, where *A* is the membrane surface.
